# Extracellular ATP mediates inflammatory responses in colitis via P2 × 7 receptor signaling

**DOI:** 10.1038/srep19108

**Published:** 2016-01-07

**Authors:** Ping Wan, Xiaopeng Liu, Yan Xiong, Yuping Ren, Jiang Chen, Nonghua Lu, Yuan Guo, Aiping Bai

**Affiliations:** 1 Department of Gastroenterology, the First Affiliated Hospital of Nanchang University, Nanchang 330006, Jiangxi Province, China; 2 Department of surgery, the Third Affiliated Hospital of Sun Yat-sen University, Guangzhou 510630, Guangdong Province, China; 3 Department of Pharmacy, the First Affiliated Hospital of Nanchang University, Nanchang 330006, Jiangxi Province, China

## Abstract

Extracellular purinergic products, particularly ATP, have recently been implicated to regulate immune cell functions and contribute to aberrant inflammatory responses of immune diseases. However, regulation of immune responses of colitis by extracellular ATP and its main receptor, P2 × 7, remains to be elucidated. In the study, we induced murine colitis by feeding mice with 4% dextran sulfate sodium (DSS), and noted dramatically heightened extracellular ATP levels in colon tissues during the progression of experimental colitis. Blockade of ATP release by carbenoxolone (CBX) treatment, or promoting ATP degradation by ATP diphosphohydrolase (apyrase), decreased extracellular ATP levels in colon tissues, attenuated DSS-induced colitis, whereas inhibition of extracellular ATP degradation by sodium metatungstate (POM-1) exacerbated tissue damage in the mice with colitis. Moreover, treatment with inhibitor of P2 × 7 receptor, A438079, decreased NFκB activation and active caspase-1 expression in lamina propria immune cells, downregulated proinflammatory cytokine production in colon tissues, and attenuated murine colitis. Collectively, these data suggest extracellular ATP participates in regulation of inflammatory responses of experimental colitis, through P2 × 7 receptor and inflammasome and NFκB signaling, which provides potential alternatives to the current clinical approaches to suppress extracellular ATP-mediated immune responsiveness.

Inflammatory bowel disease (IBD), containing two main clinical forms inclusive of Crohn’s disease (CD) and ulcerative colitis (UC), is a group of chronic intestinal disorders. Whereas etiology of IBD remains largely unknown, recent studies have noted that immune cells in intestine and colon tissues become excessively activated and contribute to progression of IBD^1^. It has been reported that immune cells including macrophages and T helper cells determine the course of disease progress^1^,^2^. After activation triggered by lumen bacterial antigens in intestine, immune cells will produce a large amount of proinflammatory cytokines e.g., tumor necrosis factor (TNF) and interleukin (IL)-1 β, and also release substantial bioactive molecules inclusive of ATP and oxygen species^2^,^3^. These cytokines and bioactive molecules further induce immune responses, resulting in sustained inflammation and damage in colon tissues^4^,^5^. Inhibition of immune cell function has been suggested as one of the crucial targets for the treatment of immune diseases including IBD^6^,^7^.

Recently, the roles of extracellular purinergic products in immune regulation are the topics of high interest. Under inflammatory conditions, once becoming activated, immune cells will release abundant ATP via ATP-releasing channels, particularly pannexin 1^8^,^9^. Injured cells also secrete ATP into extracellular milieu, resulting in high amount levels of ATP in local tissues and organs^8^. Meanwhile, ATP regulates immune cell functions through its P2 receptors^8^. Among those P2 receptors, P2 × 7 receptor is preferentially expressed by immune cells, particularly macrophages^10^,^11^. It has been reported that via P2 × 7 receptor, ATP induces a variety of bioactivities of immune cells, e.g., enhancing phagocytosis, inducing inflammasome formation, and promoting proinflammatory cytokine release^8^,^10^.

Extracellular purinergic products have been recently linked with progression of IBD. Upregulation of ecto-nucleotidase expressions has been reported in colon tissue cells of human CD patients and experimental colitis animals^12^,^13^,^14^,^15^. Meanwhile, genetic deficiency of ecto-nucleotidases in mice, which exhibit dysregulated extracellular purinergic products, has significant impact on experimental colitis^16^. Among those purinergic products, ATP is the most likely immune-regulatory molecular with capacity to regulate immune responses, and its function in IBD has been recently postulated. Administration of extracellular purines, e.g. ATP, exacerbates experimental colitis, through regulation of IL-17-producing T helper cell (Th17) responses^17^. Meanwhile, extracellular ATP mediates death of enteric neurons during colitis^18^, and participates in mast cell-dependent intestinal inflammation^19^.

Immune cells inclusive of macrophages and Th cells are the major effector and mostly pathogenic cells participating in the pathogenesis of IBD, and functionalities of those immune cells are associated with disease activity, course, and relapse^20^. However, regulation of immune cell functionalities by extracellular ATP-P2 × 7 receptor signaling and the related mechanisms remain to be elucidated. In the study, we explored the pivotal role of extracellular ATP levels and its P2 × 7 receptor signaling in the development of colitis.

## Results

### Extracellular ATP is released in colon tissues during the process of colitis

**Figure 1:**
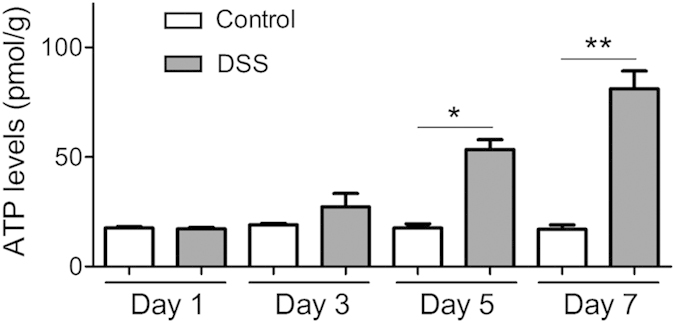
Extracellular ATP levels in colon tissues during the process of colitis. Colitis was induced by feeding the mice with distilled water containing 4% DSS since day 1 to day 8. Extracellular ATP levels in colon tissues were determined on day 1, 3, 5 and 7 (n = 4). *p < 0.05; **p < 0.01.

IBD is characterized by excessive immune cell activation and sustained tissue cell damage^21^, indicating potential of substantive ATP release into tissue milieu by activated immune cells and injured/dead cells. We thus determined extracellular ATP levels in colon tissues during the progression of DSS-induced colitis. As shown in Fig. 1, extracellular ATP levels in colon tissues of Control mice kept consistent under physical conditions, i.e., from day 1 to day 7. However, in coincidence with histological changes in colon tissues (Supplemental Fig. 1), extracellular ATP levels in colon tissues of colitis mice were dramatically increased since day 5, significantly higher than those Control mice, and reach the maximal at day 7. The data indicated significant increase of extracellular ATP levels during the process of colitis in mice.

### Blockade of ATP release abrogates DSS induced colitis

**Figure 2:**
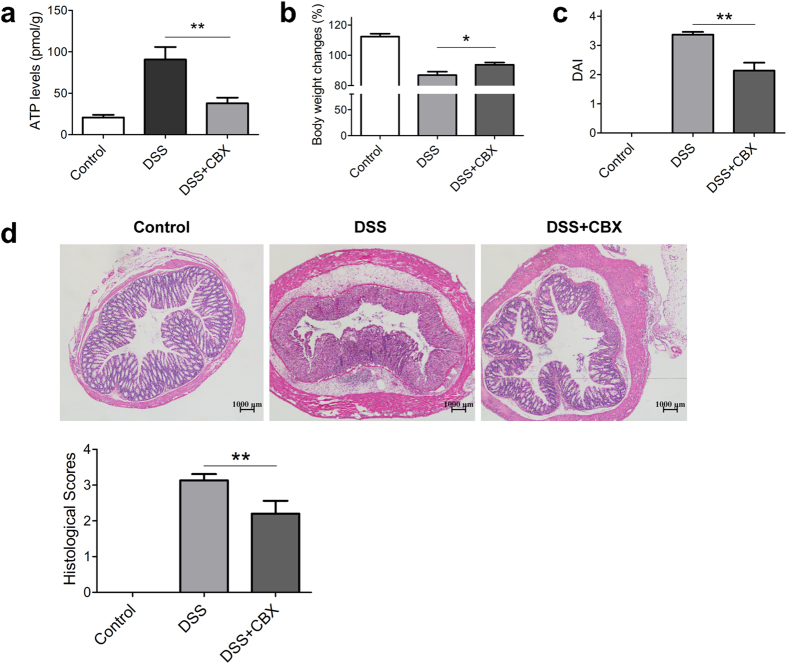
Blockade of ATP release abrogates DSS-induced colitis. The mice were fed with 4% DSS to induce colitis since day 1. CBX (50 mg/kg body weight) (DSS + CBX) or vehicle (DSS) was injected (i.p) daily into mice from day 3 to day 7. The mice were sacrificed at day 8, ATP levels in colon tissues were determined (n = 5) (**a**), body weight changes and DAI scores were recorded (n = 10) (**b,c**), respectively. HE staining was performed and histological scores were graded (n = 10) (**d**), scale bars: 1000 μm. *p < 0.05; **p < 0.01.

Upon a variety of stimulations, ATP can be released by membrane transporters, particularly Pannexin 1 (PANX1)^8^,^22^, and thus exhibit its bioactivities. To block ATP release from cells, we employed carbenoxolone (CBX), a specific inhibitor of PANX1, and noted that CBX treatment drastically decreased extracellular ATP levels in colon tissues of colitis mice (Fig. 2a), suggesting that CBX administration significantly inhibits ATP release by colon cells during the process of DSS-induced colitis.

In consistence with decreased extracellular ATP levels, management with CBX attenuated DSS-induced colon inflammation, evidenced with regained body weight (Fig. 2b), and decreased disease activity index (DAI) scores (Fig. 2c). Meanwhile, histological examination showed that DSS colitis mice exhibited severe tissue damage, whereas CBX treatment abrogated DSS-induced histological changes of colitis mice (Fig. 2d).

### Blockade of ATP degradation exacerbates tissue damage by colitis

**Figure 3:**
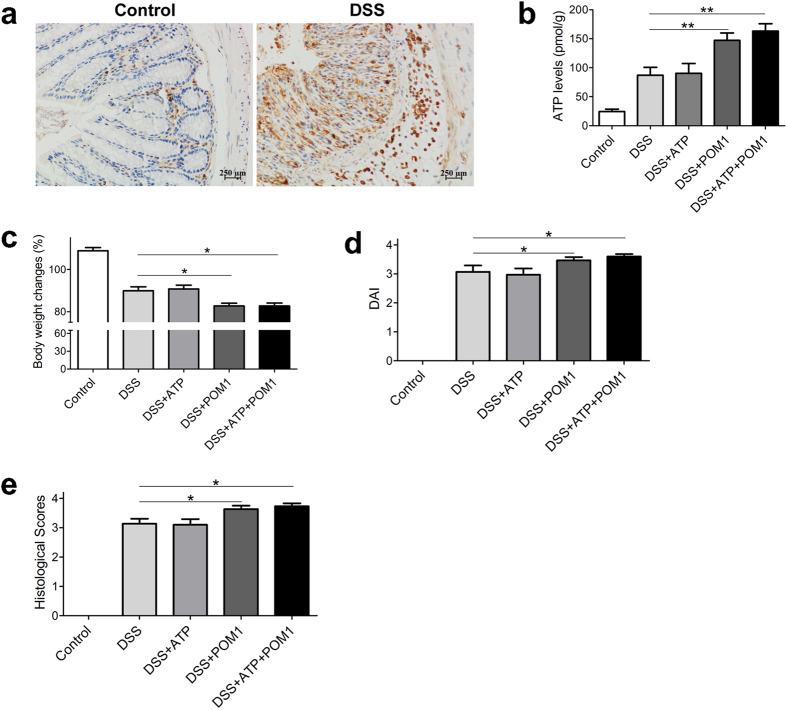
Blockade of ATP degradation exacerbates DSS-induced colitis. After induction of DSS colitis, the mice with colitis were administrated with vehicle, POM1 (10 mg/kg, i.p), or/and ATP (1 mg dissolved in 0.1 mL PBS per mouse, enema) daily since day 3 to day 7. CD39 expression was examined by immunohistochemistry before and 7 days after induction of colitis (**a**), scale bars: 250 μm. At day 8, ATP levels in colon tissues were determined (n = 5) (**b**), body weight changes (**c**) and DAI (**d**) were recorded, and histological scores were graded (**e**), respectively (n = 10). *p < 0.05; **p < 0.01.

CD39 (also known as Ecto-Nucleoside Triphosphate Diphosphohydrolase 1 or ENTPDase 1), one of ecto-nucleotidases acting to degrade ATP to ADP and AMP, is expressed by a variety of tissue cells, including endothelium, macrophages, and a proportion of T cells^23^. Because CD39 plays pivotal role in regulation of extracellular ATP levels in tissues^8^, we determined CD39 expression in colon tissues during the process of colitis, and noted up-regulated CD39 expression in colon tissues, particularly in lamina propria and submucosal layers, after induction of colitis (Fig. 3a). The data indicate the heightened CD39 expression by immune cells in lamina propria during the progression of intestinal inflammation, in accordance with the previously described observations^12^,^13^,^15^.

Owing to the potential degradation of extracellular ATP by upregulated CD39 levels in colon tissues, we next studied the effect of inhibition of CD39 bioactivity in intestinal inflammation induced by DSS. Blockade of CD39 bioactivity by sodium metatungstate (POM-1), a pharmacological inhibitor of NTPDase activity, significantly elevated extracellular ATP levels in colon tissues (Fig. 3b). Further measurements showed that, administration of POM-1 resulted in severe disease symptoms including body weight loss (Fig. 3c), and DAI scores (Fig. 3d). Simultaneously, colitis mice with POM1 treatment exhibited worse histological tissue damage in colon, in comparison with the mice of DSS colitis group (Fig. 3e and Supplemental Figure S2). The data indicated that blockade of ATP degradation by POM1 exacerbates tissue damage in mice with colitis.

### Enhancement of ATP degradation ameliorates colitis

**Figure 4:**
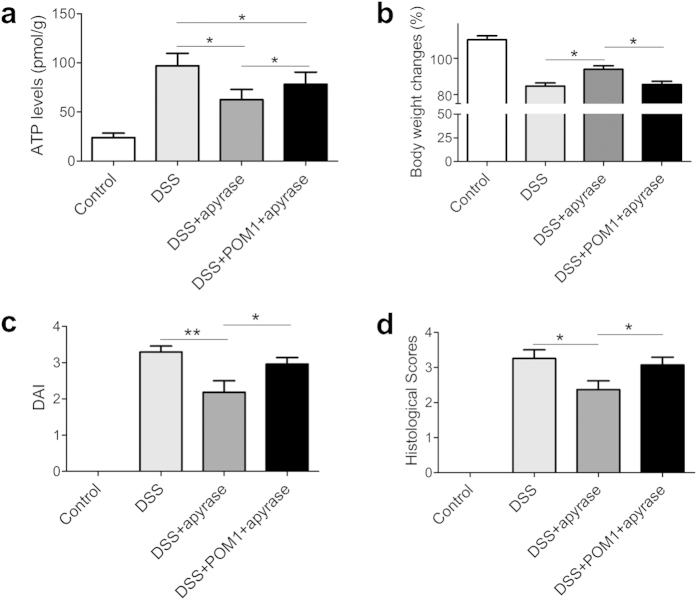
Enhancement of ATP degradation ameliorates colitis. After induction of DSS colitis, the mice with colitis were administrated i.p with vehicle, POM1 (10 mg/kg), or/and apyrase (2000 U/kg) daily since day 3 to day 7. At sacrifice of the mice at day 8, ATP levels in colon tissues were determined (n = 5) (**a**), body weight changes (**b**) and DAI (**c**) were recorded, and histological scores were graded (**d**), respectively (n = 9). *p < 0.05; **p < 0.01.

Since extracellular ATP levels are heightened in colon tissues during the process of colitis, as shown above, we next studied intestinal inflammation by induction of extracellular ATP degradation. As shown in Fig. 4a, treatment of apyrase, an ATP diphosphohydrolase, significantly decreased extracellular ATP levels in colon of mice with colitis. Intriguingly, apyrase exhibited antagonistic effect on POM1, and reduced extracellular ATP levels after its combination treatment with POM1.

Bioactivities of apyrase were further examined *in vivo*. Apyrase treatment significantly facilitated regain of body weight (Fig. 4b), decreased DAI scores (Fig. 4c), and attenuated tissue damage (Fig. 4d and Supplemental Figure S3). In concordance with extracellular ATP levels in colon tissues as shown in Fig. 4a, apyrase treatment, at least partly, counteracted POM1 bioactivity, and ameliorated POM1-induced tissue damages. The data indicate that promotion of ATP degradation by apyrase ameliorates DSS induced colitis.

### Blockade of P2 × 7 receptor alleviates colitis

DSS-induced colitis is characterized by excessive activation of immune cells, which are responsible for aberrant immune responses in colon tissues^24^. We thus hypothesized that, through its receptors, extracellular ATP regulated functionalities of immune cells and mediated inflammatory responses in colon as seen in the mice with DSS-induced colitis. Because P2 × 7 receptor is dominantly expressed by lamina propria cells and myenteric plexus^18^,^25^,^26^, and upregulation of P2 × 7 receptor in intestinal mucosa exhibits potential in the pathogenesis of Crohn’s disease^25^, we explored the mechanisms of ATP-P2 × 7 receptor signaling in regulation of inflammatory responses of experimental colitis.

**Figure 5:**
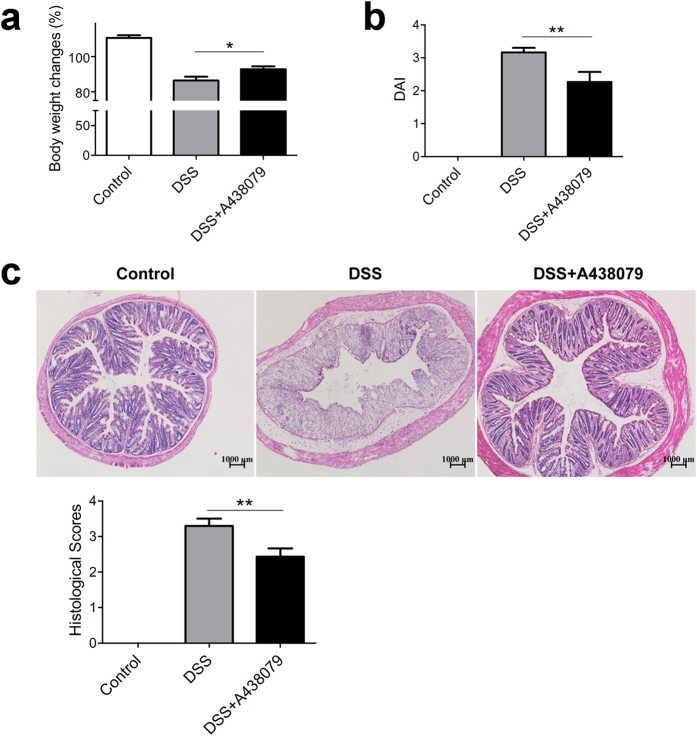
Blockade of P2 × 7 receptor attenuates DSS induced colitis. Colitis model was induced since day 1, and A438079 (100 mg/Kg) or vehicle was injected (i.p) into the mice from day 3 to day 7. After the mice were sacrificed at day 8, body weight changes and DAI were recorded (n = 10) (**a,b**), respectively. HE staining was performed and histological scores were graded (n = 10) (**c**), scale bars: 1000 μm. *p < 0.05; **p < 0.01.

We employed a specific P2 × 7 inhibitor, A438079^27^, to study the bio-functions of P2 × 7 in the progression of experimental colitis. Administration of A438079 attenuated DSS-induced colitis, as shown by improved body weight regain (Fig. 5a), reduced DAI (Fig. 5b) in DSS+ A438079 group mice, in comparison with those DSS colitis mice. In concomitant with disease symptoms, A438079 treatment alleviated DSS-induced histological tissue damage (Fig. 5c). The data indicate amelioration of experimental colitis by blockade of P2 × 7 receptor.

**Figure 6:**
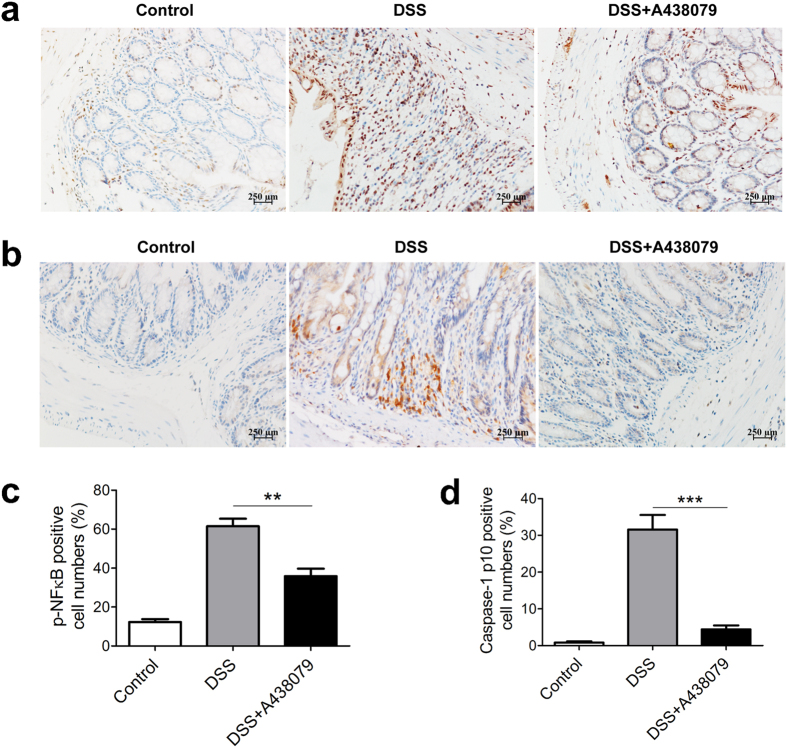
A438079 treatment down-regulates NFκB and caspase-1 activation in colon of colitis mice. Immunohistochemical staining of phosphor-NFκB p65 (**a**) and caspase-1 p10 (**b**) was performed in sections from colonic tissues of three group mice: Control, DSS, and DSS+ A438079, scale bars: 250 μm. Phosphor-NFκB p65 (**c**) or caspase-1 p10 (**d**) positive cell numbers in per 100 cells in lamina propria of the mice were counted and summarized respectively (n = 7). **P < 0.01; ***p < 0.001.

Because NF-κB has been reported as one of the pivotal factors in regulation of immune responses^28^, next we have studied whether A438079 treatment could impact on NF-κB activation. As shown in Fig. 6a,c, DSS colitis was characterized by drastic increase of phosphor-NF-κB p65 expression by lamina propria immune cells. However, administration of A438079 significantly decreased phosphor-NF-κB p65 positive cell numbers in lamina propria, suggesting inhibition of NF-κB activation by blockade of P2 × 7 receptor signaling.

P2 × 7 receptor signaling has been associated with inflammasome pathway^29^. We thus determined active caspase-1 expression, i.e., p10, a subset of caspase-1^30^, in lamina propria immune cells. During the progression of colitis, active caspase-1 expression was dramatically induced in lamina propria immune cells (Fig. 6b,d). Intriguingly, A438079 treatment significantly diminished active caspase-1 expression in colitis mice (Fig. 6b,d). The data suggest that blockade of P2 × 7 receptor inhibits inflammasome signaling and active caspase-1 expression.

**Figure 7:**
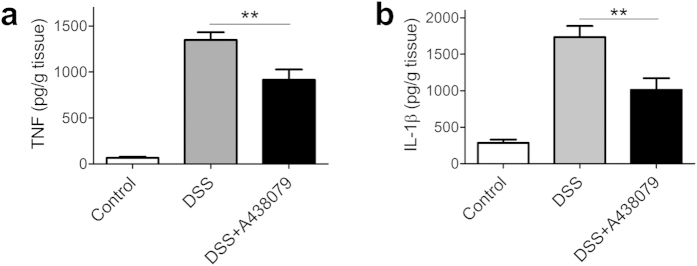
Blockade of P2 × 7 receptor decreases cytokine levels in colon tissues of mice with DSS-induced colitis. TNF (**a**) and IL-1β (**b**) levels in colonic homogenates of each group of mice were determined by ELISA (n = 7). **P < 0.01.

Expression and production of TNF and IL-1 β as the key cytokines mediating inflammatory responses in colitis^31^, is regulated by NF-κB and inflammasome pathways^32^,^33^. As shown above that NF-κB and inflammasome pathways were regulated by blockade of P2 × 7 receptor, we next determined those cytokine levels in colonic tissues. As shown in Fig. 7a,b, A438079 treatment significantly decreased levels of TNF and IL-1β in colonic tissues of the mice with colitis, in concordance with inhibition of NF-κB and inflammasome pathways in colon tissues.

## Discussion

In the present study, we discussed the pivotal role of extracellular purinergic signaling, particularly ATP-P2 × 7 receptor signaling, in regulation of inflammatory responses of experimental colitis. Our data have shown that ATP-P2 × 7 receptor signals regulate immune responses during the progression of DSS colitis, likely through mediating NFκB and inflammasome pathways.

Recently, extracellular purinergic products, e.g. ATP in particular, and their impacts on inflammatory responses and diseases become the research topic of high interest. ATP has been shown to regulate phagocytosis of immune cells, promote formation of inflammasome, stimulate cytokine secretion, mediate oxygen release, and modulate cell proliferation and apoptosis^10^,^11^. ATP plays important roles in pathogenesis of a variety of human diseases, through its P2 receptors which comprise P2X and P2Y receptors^10^,^11^.

The pathophysiological role of extracellular ATP in regulation of intestinal inflammation has been postulated. It was reported that rectal administration of ATP boosted Th17 responses and exacerbated experimental colitis^17^. Meanwhile, extracellular ATP induced death of enteric neurons during colitis^18^, and participated in mast cell-initiated intestinal inflammation^19^. In this study, we have noted that substantial extracellular ATP levels are associated with progression of DSS colitis. Our further studies have showed that inhibition of ATP release by pharmacological inhibitor of ATP transporters including PANX1, i.e. CBX, dampens extracellular ATP levels, resulting in amelioration of DSS-induced colitis. The data above show the correlation of extracellular ATP levels with inflammation in colon tissues, indicating the particular role of extracellular ATP levels in pathogenesis of DSS-induced colitis. Meanwhile, we noted that CBX exhibited, likely to less extent, impacts on body weight regain, as compared with other parameters inclusive of extracellular ATP levels in colon and DAI, possibly indicating systematically biological effects of CBX or/and inhibition of ATP transporters.

We and others have previously demonstrated that ectonucleotidases, particularly CD39, can regulate extracellular purinergic product levels inclusive of ATP, and thus impact immune cell functionalities and immune responses^12^,^15^,^34^. CD39 sequentially hydrolyzes extracellular ATP and ADP to AMP, and the latter is ultimately degraded to adenosine by CD73/ecto-5′-nucleotidase^35^,^36^. This process is termed purinergic signaling. We next associated extracellular ATP levels regulated by purinergic signaling with intestinal inflammation. Using POM1, the well used inhibitor of CD39, we noted that blockade of CD39 bioactivity restored substantial extracellular ATP levels in colon tissues, and exacerbated intestinal inflammation of DSS-induced colitis. Nevertheless, promotion of ATP hydrolysis by apyrase, a soluble chemical with enzymatic activity identical to CD39, drastically decreased extracellular ATP levels, and attenuated experimental colitis. The data above addressed dynamic change of extracellular ATP, inhibition of ATP degradation in particular, in mediating the development of DSS-induced colitis. Meanwhile, we noted that rectal administration of ATP had minimal effect on DSS-induced colitis model. Given that DSS-induced colitis/inflammation drastically enhances the expression of CD39 in colon tissues, it is postulated that increase of CD39 levels boosts degradation of extracellular ATP, and counteracts, at least partly, the bioactivities of exogenous ATP.

Inflammasome signaling has been linked with inflammation and implicated in immune diseases^37^. Upon a variety of stimulations of immune cells, inflammasome becomes activated, leading to cleavage of pro-caspase-1 and release of active caspase-1 from inflammasome complex^30^. Active caspase-1 comprises two heterodimers inclusive of p20 and p10 which can act to catalyze cytokine maturation process inclusive of IL-1β and IL-18^30^. In this study, we have noted that DSS-induced colitis is characterized by drastic increment of active caspase-1 expression in lamina propria immune cells, whereas blockade of P2 × 7 receptor by its pharmacological inhibitor dampens active caspase-1 levels in lamina propria of colitis. The data indicate that P2 × 7 receptor signaling regulates immune responses via mediating inflammasome pathway.

NFκB is one of important transcription factors controlling immune cell functions and inflammatory responses of immune diseases^28^. Upon stimulations of lipopolysaccharides (LPS) and other extracellular bacterial antigens, the dimer of NFκB composed of the P65 and P50 subunits translocates to nucleus of immune cells, and regulates various target gene expression including a large amount of inflammatory cytokines and chemokines, resulting in sustained inflammatory responses and tissue damage^28^,^38^. Inhibition of NFκB activation and signaling can attenuate immune responses, and ameliorate immune diseases in animal models^6^,^39^,^40^, which supports the beneficial effects of inhibition of NFκB signaling. In the present study, we have found that blockade of P2 × 7 receptor signal by A438079 inhibits NFκB activation in lamina propria immune cells, which subsequently results in inhibition of immune responses and TNF levels, a key proinflammatory cytokine for the pathogenesis of IBD^41^.

Finally, we demonstrate that regulation of extracellular ATP levels in tissues abrogates immune responses of DSS-induced colitis. Under physical conditions, extracellular ATP levels are responsible for functionalities of a wide variety of cells, participating in regulation of cell activation, proliferation, and death^42^,^43^. However, when excessively released, the heightened extracellular ATP levels induce numerous pathological responses, and impact on human diseases such as central nervous system diseases and cardiovascular disease^44^,^45^. Here we show that treatment with pharmacologically active reagents, particularly CD39 analogs and P2 × 7 receptor antagonists to decrease extracellular ATP levels or/and block ATP/P2 × 7 receptor signaling, diminishes immune responses in DSS-induced colitis, perhaps indicating that regulation of extracellular ATP levels and ATP/P2 × 7 receptor signaling could be explored as a potential therapeutic target.

Collectively, these data taken together suggest associations of extracellular ATP levels and signaling with progression of experimental colitis. We also demonstrate extracellular ATP participates in regulation of inflammatory responses of colitis, through P2 × 7 receptor and inflammasome and NFκB signaling. Our findings help improve the understanding of the molecular control of ATP-P2 × 7 receptor signaling and immune responses, provide possible new alternatives to the current clinical approaches to suppress extracellular ATP-mediated immune responsiveness.

## Experimental Procedures

### Animals

Female BALB/c mice were provided by the Experimental Animal Center of Nanchang University, and fed under specific pathogen-free conditions. 7 to 8 week-old mice were used in the study, weighing approximately 22 g. All protocols for the projects using mice were reviewed and approved by the Institutional Animal Care Committee of Nanchang University. Animal care, use, and treatment were in accordance with the guidelines and regulations.

### Induction of dextran sulphate sodium (DSS) colitis and treatment of the mice

Acute DSS colitis was induced in BALB/c mice according to the previously published method with minor modification^46^,^47^. The mice were fed 4% (w/v) DSS (molecular mass, 36–50 kDa; MP Biomedicals) dissolved in the drinking water on day one. Fresh DSS solution was provided every other day. Control mice drank only distilled water. Disease symptoms of colitis were assessed daily by measurement of body weight, evaluation of stool consistency, and detection of bloody stools. Disease severity was scored using a clinical disease activity index (DAI) ranging from 0 to 4, calculated as previously described^46^ using the following parameters: stool consistency, presence or absence of fecal blood and weight loss. The mice were killed on day eight, and the middle section of colon was fixed in 10% formaldehyde–saline. Hematoxylin and eosin stain (HE)-stained sections were graded based on a scoring system modified from a previous study^48^,^49^. Histology scoring was performed, and a combined score of inflammatory cell infiltration and tissue damage was determined as follows: score 0, normal colonic mucosa and occasional inflammatory cells in the lamina propria; 1, loss of one-third of the crypts; 2, loss of two-thirds of the crypts; 3, the lamina propria is covered with a single layer of epithelium and mild inflammatory cell infiltration is present; and 4, erosions and transmural extension of infiltrate.

The mice were administrated with various reagents from day 3 to day 7. The doses of the reagents were used according to the related literatures^16^,^17^,^50^,^51^. DMSO was used to dissolve A438079 (Santa Cruz Biotechnology), and other reagents (from Sigma-Aldrich) were dissolved in saline.

### Measurement of extracellular ATP levels in colon tissues

Colon tissues were weighted right after sacrifice of the mice, and then kept in PBS containing penicillin (200 U/ml), streptomycin (200 μg/ml), and gentamycin (10 μg/ml) for 5 minutes. After three times of washing, the tissues were incubated in 10 times volume (w/v) of PBS at 37 ^o^C for 1 hour. The supernatants were collected for determination of ATP concentrations herein.

ATP levels in the supernatants were determined according to the instruction of Luminescence ATP Detection Assay System (PerkinElmer, Waltham, MA, USA) with minor modification. In brief, 50 μl of supernatants were added into each well, followed by introduction of 50 μl of the substrate solution. After shaking in dark for 10 min, luminescence intensity of each well was determined.

### Enzyme-linked immunosorbent assays (ELISA)

Tumor necrosis factor (TNF) and interleukin (IL) 1β levels in homogenates of colon tissues were determined by ELISA, following manufacturer’s instructions (R&D Systems, Inc). Briefly, polyclonal anti-mouse cytokine antibodies were used as capturing antibodies and biotinylated polyclonal anti-mouse cytokine antibodies for detection, and the standard curve of cytokines was set up meanwhile. Color changes were determined at 450 and 540 nm, respectively. The final readings were made after subtraction of readings at 540 nm from the readings at 450 nm.

### Immunohistochemistry

Colon tissues of mice were taken and fixed immediately in 10% buffered formalin, embedded in paraffin, and cut into 4 μm sections. After blockade of inner peroxidase, sections were incubated sequentially with the first antibody solution including rabbit anti-caspase-1 p10 antibody (M-20, from Santa Cruz Biotechnology), anti-NF-κB p65 (phospho S536, from Abcam) antibody, or anti-CD39/ENTPD1 MAb (Clone 495826, from R&D). After three washes in PBS (pH 7·4), the sections were then incubated in secondary goat anti-rabbit immunoglobulin (Ig)G conjugated with peroxidase labeled polymer, prior to colorization using diaminobenzidine reaction and counterstained with haematoxylin. Negative controls were established using rabbit IgG instead of the first antibodies. The sections were evaluated using light microscopy, and 100 cells in lamina propria per high power field were calculated for statistical analysis.

### Statistical analysis

All data in the text and figures are expressed as mean ± standard deviation. Comparisons of more than two groups were made with a one-way analysis of variance using Tukey’s *post hoc* test. When appropriate, comparison with two groups was made using Student’s *t*-test for unpaired data. Differences were considered statistically significant if *P*  0.05.

## Additional Information

**How to cite this article**: Wan, P. *et al.* Extracellular ATP mediates inflammatory responses in colitis via P2×7 receptor signaling. *Sci. Rep.* **6**, 19108; doi: 10.1038/srep19108 (2016).

## Supplementary Material

Supplementary Information
