# Supplementary material for: Extracellular ATP mediates inflammatory responses in colitis via P2 × 7 receptor signaling
Source: Sci Rep. 2016 Jan 7;6:19108. doi: 10.1038/srep19108 (PMC4703960; doi:10.1038/srep19108)
Supplement: Supplementary Information [file srep19108-s1.pdf]

Extracellular ATP mediates inflammatory responses in colitis via P2X7 receptor signaling

Ping Wan, Xiaopeng Liu, Yan Xiong, Yuping Ren, Jiang Chen,

Nonghua Lu, Yuan Guo, Aiping Bai

Supplemental Figure legends

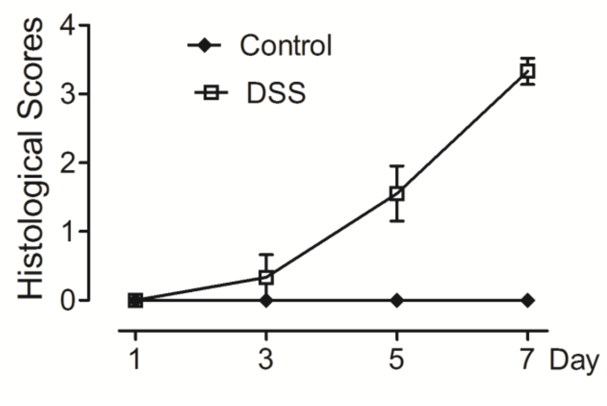

Supplemental Figure 1. Histological scores of colon tissues during the process of colitis. Colitis was induced by feeding the mice with distilled water containing 4% DSS since day 1 to day 8. Histological changes in colon tissues were determined on day 1, 3, 5 and 7 (n=3).

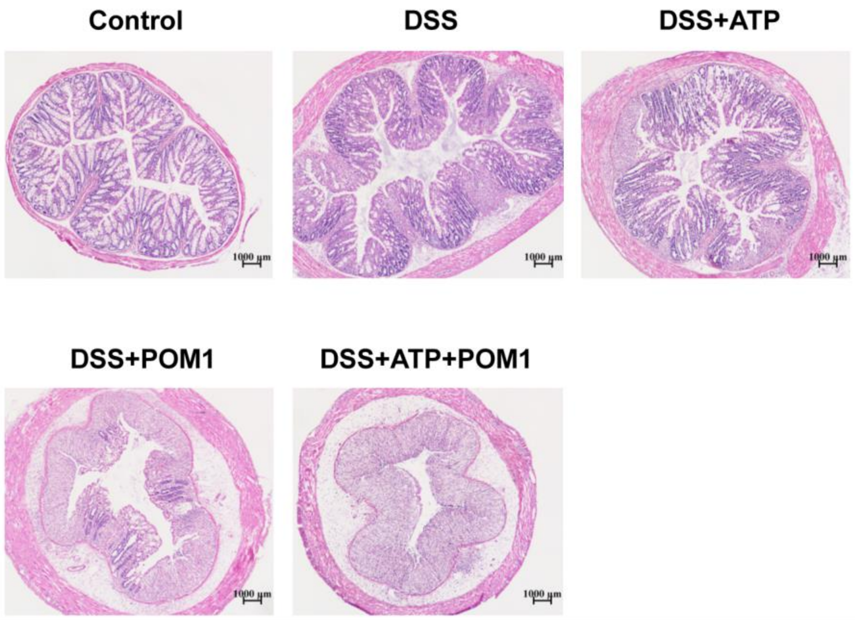

Supplemental Figure 2. Representative hematoxylin and eosin staining of colons from five group mice: Control, DSS, DSS+ATP, DSS+POM1, and DSS+ATP+POM1. Scale bars: 1000 μm.

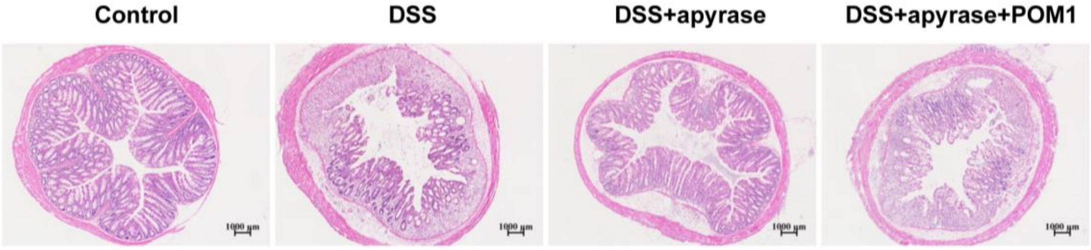

Supplemental Figure 3. Representative hematoxylin and eosin staining of colons from four group mice: Control, DSS, DSS+apyrase, and DSS+apyrase+POM1. Scale bars: 1000 μm.
